# Analyzing factors affecting risk aversion: Case of life insurance data in Korea

**DOI:** 10.1016/j.heliyon.2023.e20697

**Published:** 2023-10-05

**Authors:** Sehyun Lim, Taeyeon Oh, Guy Ngayo

**Affiliations:** a Seoul Business School, aSSIST University, 6 Ewhayeodae 2-gil, Fintower, Sinchon-ro, Seodaemun-gu, Seoul, South Korea, 03767; b Seoul AI School, aSSIST University 6 Ewhayeodae 2-gil, Fintower, Sinchon-ro, Seodaemun-gu Seoul, South Korea, 03767; c Franklin University Switzerland, Via Ponte Tresa 29, 6924 Sorengo, Switzerland

**Keywords:** Risk aversion, Life insurance, Private health insurance, Big data, Real-world data (RWD), Machine learning, Stacking ensemble model

## Abstract

This research employs machine learning analysis on extensive data from a prominent Korean life insurance company to substantiate the insurance demand theory, which posits that insurance demand increases with risk aversion. We quantitatively delineate the traits of risk-averse individuals.

Our study focuses on a cohort of 94,306 individuals who have filed insurance claims due to illness. To forecast prospective insurance consumers inclined toward additional purchases, we construct a predictive model using a machine learning algorithm. This model incorporates 19 demographic and socioeconomic factors as independent variables, with additional insurance acquisition as the dependent variable. Consequently, we uncover the distinctive characteristics of consumers predicted to acquire supplementary insurance products.

Our findings reveal a significant association between the independent variables and the likelihood of purchasing additional insurance. Notably, 10 out of the 19 independent variables exert a substantial influence on additional insurance acquisitions. These characteristics encompass residence in rural areas, a higher likelihood of being female, advanced age, increased assets, a higher likelihood of being blue-collar workers, lower education levels, a greater likelihood of being married or divorced/separated, a history of cancer, and a predisposition for existing policyholders with prior subscriptions to actual loss insurance or substantial insurance contract amounts.

Our study holds academic significance by addressing limitations observed in prior research, which predominantly relied on questionnaires to qualitatively assess risk aversion. Instead, we offer specific insights into individual characteristics associated with risk aversion.

Moreover, we anticipate that Korean insurance companies can leverage these insights to attract new clientele while retaining existing members through predictive risk aversion analysis. These findings also offer valuable insights across a spectrum of disciplines, including business administration, psychology, education, sociology, and sales/marketing, related to individuals' risk preferences and behaviors.

## 1 Introduction

Growth rate of new contracts in Korea's life insurance industry has been decreasing over time, especially since the 1980s when it experienced a period of rapid expansion [58]. Additionally, for life insurance specifically, the number of contracts held per household has reached a saturation point [1]. As a result, life insurance companies in Korea have shifted their focus from acquiring new contracts to retaining existing customers while exploring avenues to attract additional subscriptions.

Life insurers in Korea are granted authorization to offer both life insurance, which provides benefits in the event of death or permanent disability due to various causes, and health insurance, primarily designed to cover medical expenses. This dual offering allows existing policyholders to tailor their insurance coverage to meet their individual needs, effectively preparing for a range of life and health-related risks, including diseases and accidents.

While financial consumers are expected to make objective and rational decisions regarding their finances, their attitudes towards money are surprisingly influenced by emotions [2]. Psychological factors, such as the propensity to control savings and spending, interest in money-related knowledge, risk tolerance, and the need for advice from others, play a significant role in financial decision-making. The UK's Financial Supervisory Service has even highlighted that psychological factors have a greater impact on consumer financial behavior than economic or financial information [3].

Given that insurance is primarily a means to mitigate future risks, psychological factors are likely to strongly influence the purchasing behavior of insurance consumers, alongside economic factors. Previous studies have shown that consumers who are risk-averse are more inclined to seek stability and are more likely to purchase insurance products as a way to prepare for future risks [4]. Additionally, factors such as work ethic, religion, and education have been identified as important determinants of life insurance ownership [5]. These findings emphasize the close relationship between psychological factors and insurance attitudes, underscoring the need to identify psychological factors that impact insurance subscription behaviors.

To empirically investigate these factors, this study focuses on the number of additional insurance products subscribed by policyholders of a major insurance company ‘A’ in Korea.

In line with insurance demand theory ([27,28,42]), which posits that individuals in poor health are more inclined to mitigate risk by purchasing additional insurance, our analysis focuses on policyholders who made claims due to illness. We examined whether customers of insurer 'A' who held policies from 2019 to 2022 increased their policy coverage in subsequent years following illness-related claims before 2020. The analysis revealed a significant difference, with customers who had a history of illness-related claims showing a 7.8% point higher percentage of customers with increased insurance contract amount, approximately 1.87 times higher, compared to customers without such claims, as shown in Table 1.

**Table 1:** Analysis of the increase and decrease of the insurance contract amount before and after 2020 according to whether the customer had a claim history from 2019 to 2022.

	Number of Customers who held policies from 2019 to 2022	Difference in insurance contract amount before and after the reference point of Y2020	
		Keep or Decrease (%)	Increase (%)
Claim Occurs	46,250	83.3 %	16.7 %
No Claim	614,016	91.1 %	8.9 %

In order to assess individuals' perceptions of risk, the primary focus is on the dependent variable, which pertains to the presence or absence of a track record of insurance claims linked to health-related issues. Additionally, the study encompasses a selection of nineteen independent variables encompassing gender, age group, income status, marital condition, number of children, educational attainment, affluence tier, occupation, and type of illness claimed.

Using the Orange open-source software (version 3.34), the prediction model is developed using machine learning algorithms such as Random Forest and Gradient Boosting to identify the most influential variable in predicting additional insurance subscriptions. This approach allows for the quantification of consumers' risk aversion through objective quantitative information, such as age, gender, and health status, rather than relying solely on subjective measures like risk aversion questionnaires. The aim is to provide insights into the specific demographic and socioeconomic characteristics of consumers that indicate a propensity for risk aversion.

This study differs from previous research in two key aspects. Firstly, it utilizes big data collected from the life insurance company in Korea to overcome the limitations of previous research relying on surveys with sample size constraints and potential biases. Secondly, it provides a machine learning-based predictive model that predicts the impact of individual characteristics representing risk aversion on insurance subscription.

The determinants of risk attitudes are a universal subject of study across various disciplines, including business administration, psychology, education, and sociology. The field of behavioral finance, which focuses on the psychological characteristics influencing individuals' financial practices, has gained significant attention. Therefore, this study aims to bridge the gap between human psychology and behavior by identifying specific influencing factors that impact the risk aversion of financial consumers. The determinants of risk attitudes explored in this study have wide-ranging implications for insurance companies seeking to enhance relationships with existing customers through additional product subscriptions. Consequently, insurance companies can use the predictive model presented in this study to strengthen their relationship with customers in a customized way according to individual characteristics. In addition, the variables that influence consumer risk aversion derived from this study provide meaningful insights into academia, industry, and policy decisions.

The rest of this research is arranged as follows. Section 2 introduces a literature review describing the relationship between insurance demand and explanatory variables that influence risk aversion. Section 3 presents the method of data collection and the predictive model used. Section 4 selects a predictive model that exhibits best performance and shows the relationship between important variables and risk aversion of customers. Section 5 discusses the results. Section 6 discusses the implications of this research. Finally, Chapter 7 concludes this study and presents significance and limitations of our findings.

## 2 Literature review

### 2.1 Insurance demand and socioeconomic influencing factors

Numerous prior studies have investigated the relationship between insurance consumers' economic circumstances and their insurance contract subscriptions, often highlighting a positive correlation between income or assets and insurance purchases. For instance, Xiao [6] examined the variations in insurance assets based on household income and found that higher income levels corresponded to a greater probability of purchasing life insurance. Similarly, the study conducted by Bernheim [7] revealed a strong positive correlation between insurance purchases and total wealth. Additionally, Hau [8] confirmed a positive association between net assets and life insurance purchase and between pension assets and life insurance purchases.

In tandem with these investigations, researchers have also explored the impact of macroeconomic indicators, closely tied to opportunity costs, on policyholders' insurance subscription behavior. Several studies, such as Dar and Dodds [9], Outreville [10], and Hau [8]), have examined the effects of interest rates and unemployment rates on insurance contract retention. Subsequently, Kim [11], Cox and Lin [12], and Kiesenbauer [13] expanded this line of inquiry to evaluate the relationship between additional economic indicators, including gross domestic product (GDP) and capital market development, and insurance policy retention. Kagraoka [14] discovered that the number of insurance cancellations increased in tandem with rising unemployment rates after the insurance contract date, implying a strong linkage between insurance contract cancellations and changes in unemployment rates. Similarly, Kiesenbauer [13] confirmed that insurance policy cancellation rates decreased during periods of economic prosperity, characterized by an increase in GDP and a decrease in the unemployment rate.

However, it should be noted that research results have also demonstrated the complexity of explaining insurance policyholders' maintenance and withdrawal solely based on economic factors. For instance, Anderson and Nevin [15] observed a positive relationship between current income and life insurance demand in low-income and high-income households but found a negative relationship among middle-income families. Similarly, Berekson [16]'s survey of college students revealed contradictory findings. While one university's sample exhibited a strong positive relationship between life insurance demand and current income, another university's sample showed no significant correlation.

Moreover, Di Matteo and Emery [17] analyzed the relationship between assets and life insurance needs and discovered that individuals with fewer accumulated assets displayed a higher demand for life insurance during the early stages of the life cycle. This finding suggests that the influence of asset size on insurance behavior varies depending on the individual's circumstances.

Even when examining the results of a survey conducted by the Korea Life Insurance Association [18] on households that had canceled life insurance contracts, it is challenging to discern a consistent pattern regarding the level of insurance premium payments relative to household income and the average duration of life insurance contracts. The study confirmed a low correlation between burden capacity and insurance contract duration, indicating that the decision to maintain or terminate an insurance contract may not be solely driven by the insured individual's economic factors.

### 2.2 Insurance demand and risk aversion factors

Perceived risk, defined by Slovic [19] as an individual's subjective judgment regarding the likelihood of an adverse event, elicits varied responses among individuals in similar risky situations. To elucidate the profiles of risk-preferring and risk-averse individuals, several psychological studies have been conducted.

Kogan and Wallach [20] discovered that differences in behavior among individuals facing similar risky situations could be partially explained by factors such as family background, education, and occupational position. In an analysis of risk-seeking propensity among Korean investors, Min and Song [21] found that males and individuals with higher annual incomes exhibited a relatively higher inclination towards risk-taking. From the perspective of consumer financial management, Jang and Choe [22] revealed that consumers with a low level of objective financial understanding and no investment experience displayed characteristics associated with risk aversion. Furthermore, Joung and Shin [23] indicated that individuals with male, younger, unmarried status and a higher self-confidence tended to exhibit higher levels of risk tolerance.

In the realm of health economics, numerous studies have explored the relationship between risk avoidance tendencies and personal health management activities, as well as psychological factors such as depression or anxiety symptoms. Anderson and Mellor [24] provided evidence that risk-averse individuals consistently engaged in fewer health-related risky behaviors such as smoking, excessive drinking, obesity, and failure to use seat belts. Similarly, Cutler and Glaeser [25] confirmed a correlation between risk preference and engagement in activities that can harm one's health. Yoo and Kwon [26] observed that a temperament of risk avoidance resulted in increased attempts to avoid experiences and heightened worry symptoms.

Considering the perceptual aspects, it is concluded that as the psychological risk of negative events increases, so does the demand for insurance as a risk hedge, even with the same premium. Consequently, studies aiming to identify psychological factors influencing insurance demand have primarily focused on risk aversion. In the 1960s, Yaari [27] and Hakansson [28] developed an insurance demand theory suggesting that individuals with higher risk aversion exhibit a greater demand for insurance. Subsequent studies, including Schlesinger [29] and Szpiro [30], followed suit.

Dionne and Eeckhoudt [31] found that higher levels of risk aversion were associated with a preference for insurance, a financial product offering low risk and low loss. Examining risk aversion in conjunction with the wealth effect, Chesney and Loubergé [32] concluded that the two factors were interdependent, emphasizing the need to consider both risk aversion and wealth when determining their impact on insurance demand. Lee [33] investigated the intention to purchase accident insurance and found that media exposure positively influenced new purchase intentions, partially mediated by negative risk sentiment (fear and worry) regarding traffic accidents.

Initially, correlation analysis between risk aversion, a psychological variable, and insurance subscriptions was conducted using experimental economics due to a lack of empirical data to support the theory ([[34], [35], [36]]). Subsequent studies focused on measuring individuals' psychological risk perception through questionnaires, as quantifying risk avoidance, a psychological variable, proved challenging. Lease, Lewellen [37] attempted to measure individual risk aversion by employing a comprehensive 130-question survey. However, the study's limited sample size of 990 out of 3000 subjects hinders the representativeness of the findings. Grable and Lytton [38] measured individual risk tolerance using a 20-item questionnaire, including inquiries such as "Do your friends consider you a risk-taker?" and "What word comes to mind first when you hear 'risk': 'loss,' 'uncertainty,' 'opportunity,' or 'thrill'?" Nevertheless, the analysis results are not widely generalizable due to the use of self-reported questionnaires and an in-house sample. Consequently, surveying individuals' risk aversion propensity, as in "What is your risk aversion propensity?", may encounter limitations related to bias.

To substantiate the insurance demand theory, attempts have been made to replace direct survey results for individual risk aversion with general demographic and socioeconomic variables. Age, gender, income, education level, and marital status have been utilized as explanatory variables to examine their relationship with life insurance premiums, analyzing which factors determine the degree of risk aversion. These studies share several common characteristics. Firstly, they predominantly employ life insurance premiums as the dependent variable, as life insurance is voluntarily purchased, contrasting with non-life insurance encompassing compulsory or policy insurance. Secondly, previous studies have employed demographic and socioeconomic variables highly associated with risk aversion as proxy variables, instead of relying on a single variable representing risk avoidance ([39,40]).

Burnett and Palmer [5] revealed that life insurance consumers tended to be non-opinion leaders, risk-averse, less price-sensitive, possess low self-esteem, exhibit low assertiveness, distrust fate, show disloyalty to brands, display less reliance on government, and maintain stable residences. Incorporating psychological variables alongside traditional demographic factors enhanced the model's explanatory power in predicting life insurance demand. Kim&Jung [1] considered age, gender, economic status, insurance service benefits, and willingness to purchase insurance as independent variables influencing individual insurance contract maintenance. Lee, Kwon [41], utilizing 2005 survey data from the Korea Insurance Development Institute (KIDI), investigated the impact of residential areas categorized into large cities, small cities, and local areas and job grades on insurance demand.

Moreover, among several independent variables highly associated with risk aversion among insurance consumers, consumer disease information has been studied as a crucial determinant of insurance demand. Given that protection-type insurance products ultimately provide insurance coverage for injuries or illnesses, consumers experiencing fatigue or abnormal symptoms may seek to mitigate risk by purchasing insurance independently, without relying solely on marketing efforts or information dissemination. Consequently, insurance companies endeavor to gather health information from insurance consumers through compulsory health examinations or questionnaires prior to insurance subscription, aiming to equip consumers (i.e., insurance subscribers) with risk perception information about their own vulnerabilities. This serves as a defense against the "adverse selection" phenomenon in insurance demand, which assumes that policyholders possess more asymmetric information than the insurer.

The potential for such "adverse selection" in insurance demand is rooted in theoretical models from Rothschild and Stiglitz [42], Yaari [27] and Hakansson [28] studies, highlighting the direct influence of consumer disease information on insurance demand and its indirect effects through risk perception. Rosenberg and Zhong [54] studied U.S. consumers and found that those with the highest healthcare expenditures had the highest rates of health insurance enrollment compared to other groups. Lee [33] demonstrated a statistically significant correlation between insurance purchase intention, as the dependent variable, and the likelihood of underlying disease, serving as an independent variable. Kim [43] analyzed that individuals who perceived a higher risk of developing cancer in Korea were 26.3% points more likely to subscribe to cancer insurance compared to those perceiving a lower risk.

However, previous studies encountered challenges in establishing consistent correlations between individual characteristics and risk aversion, often revealing contradictory effects of each explanatory variable on risk aversion. For instance, Dohmen, Falk [44] found a significantly positive effect of high parental education on willingness to take risks, while Jianakoplos and Bernasek [45] asserted that risk aversion increased with the level of education. These findings underscore the incongruity between an individual's level of education and their risk aversion.

Similarly, Outreville [46] identified that wealthy individuals with high levels of education exhibited lower risk aversion, aligning with Dohmen, Falk [44]'s findings, yet less risk-averse individuals tended to have higher levels of education. Outreville [46] proposed that this outcome may result from a deliberate choice for less risk averse individuals to pursue higher level of education.

Moreover, previous studies investigating the effect of each explanatory variable, such as age, education, and marital status, on insurance demand revealed inconsistent correlations (see Table 2). These inconsistencies can be attributed to limitations in sampling, impacting representativeness and introducing bias.

**Table 2:** Demographics associated with the demand for life insurance: Examples of priori studies.

Authors	Data	Variables
Hammond, Houston [36]	US household survey. 1952 and 1961	Age (NS), Education (+),Marital status (−), Family size (−), Race (n.s)
Berekson [16]	College student survery	Age (+), Marital status (NS), Family size (+)
Ferber and Lee [47]	Married Couple interviews	Age (−), Education (+), Family size (+)
Showers and Shotick [48]	Consumer Expenditure Survery, 1987	Age (+), Family size (+)
Gutter and Hatcher [49]	Survey of Consumer Finance, 2004	Age (+), Education (−), Family size (n.s), Race(n.s)

Note: n.s indicates the statistical insignificance.

Overall, while numerous studies have explored the relationship between risk aversion, individual characteristics, and insurance demand, the inconsistent findings and methodological limitations in previous research highlight the need for further investigation to enhance our understanding of these complex dynamics.

## 3 Quantitative analysis

### 3.1 Datasets

The research data set in this article was extracted from a large life insurance company ‘A’ in Korea, which had a customer base of 4,003,914 individuals as of October 2022. This company ranked third among Korean life insurance companies in terms of the number of contracts held (8,975,000 out of 87,099,000 in Korea, accounting for 10.3 %), making it a suitable representation of the general insurance consumer population in the Korean life insurance market.

To predict customers with risk aversion, the dataset included insurance customers who subscribed to insurance after 2000 and had a history of insurance claims due to illness between 2018 and 2020. Claims arising from illnesses encompass a spectrum of benefits, comprising medical procedures like hospitalization and surgery within life insurance policies, along with provisions within health insurance policies pertaining to medical expenses. In an effort to mitigate the phenomenon of "adverse selection" prevalent in insurance procurement, we applied a set of stringent criteria: prospective policyholders needed to fall within the age bracket of 19–59, a range where more favorable underwriting is typically observed, maintain their insurance contract for a minimum duration of 12 months post-claim submission, and possess an active insurance contract as of October 2022, which served as the date for data extraction. Additionally, customers with the same policy holder and insured were included to utilize disease onset data for identifying individual risk aversion tendencies. The final dataset consisted of 94,306 customers from Company A who met all the specified conditions.

#### 3.1.1 Dependent variable

In this study, we adopted a research method to examine the individual's propensity for risk aversion as the dependent variable. To quantify this propensity, we focused on the additional purchase of insurance among research subjects who had a history of insurance claims due to illness. The presence of supplementary insurance is represented as a discrete dichotomous outcome variable, and we employed a binary outcome variable, consistent with the approach employed by Shi and Shi [55], wherein the variable assumes a value of 1 to denote the existence of one or more instances of supplementary insurance and 0 to signify its absence.

To be more specific, the cases where customers had claimed insurance for illness between 2018 and 2020 was specifically examined, and we focused on assessing whether these customers had a history of additional insurance subscriptions by the end of the year following the month of their insurance claim. The clients' supplemental insurance histories included both life and health insurance products that they could purchase as needed based on their individual circumstances and priorities. The rationale behind this specific time frame is to ensure consistency in evaluating the outcomes of additional subscriptions. By applying the same period until the end of the year following the insurance claim month, we aimed to mitigate variations that could arise from different criteria used to determine additional subscriptions after the insurance claim month. For instance, customers billed in 2018 could be evaluated for additional subscriptions over a period of approximately 3.5 years, from the time of billing until December 2021. On the other hand, customers billed in 2020 would be assessed for additional subscriptions over a period of about 1.5 years, from the time of billing until December 2022. This approach enables us to account for potential discrepancies in the number of additional subscriptions resulting from differences in billing years, thus avoiding the manifestation of distinct characteristics solely based on the billing year. By implementing these standardized criteria for the dependent variable, we sought to proactively control potential confounding factors. As a result, the number of customers who claimed insurance for illness between 2018 and 2020 by year is shown in Table 3 below.

**Table 3:** A year-by-year comparison of the number of customers making claims and adding insurance.

Claim Year	2018	2019	2020
Number of customers with claims	35,793	32,651	25,862
Supplemental Insurance Period	Billing date - Dec 2020	Billing date - Dec 2021	Billing date - Oct 2022
Number of customers with additional insurance	7050	7167	5779
Share of additional insurance	19.70%	21.95 %	22.34 %

Consequently, Table 3 displays the annual distribution of customers who filed insurance claims for medical purposes between 2018 and 2020. Over the three-year period, there was a discernible upward trajectory in the proportion of customers opting to acquire supplementary insurance products. This phenomenon can be attributed to the assessment timelines for customers who filed claims in 2018 and 2019, which extended until December of the subsequent year, in contrast to the assessment window for those who made claims in 2020, which concluded at the end of October. The plausible explanation for this observed trend is the growing inclination among insurance consumers to mitigate their risks through supplementary insurance, a phenomenon potentially fueled by the onset of the COVID-19 pandemic in 2020.

#### 3.1.2 Independent variable

In this study, we employed a comprehensive set of 19 independent variables to investigate their relationship with individual risk aversion. These variables were carefully selected based on their relevance to the research objective and the ability to capture the comprehensive risk profile of the target company's insured individuals. A summary of these variables can be found in Table 4.

**Table 4:** Configuration of variables.

	explanatory variable	Variable characteristics
Dependent variable	Additional insurance subscription status	Categorical type (No subscription:0, Subscription:1)
Independent variables	Gender	Categorical type (Female:0, Male:1)
	Age	Categorical type (under30s:lower30, 30s:Age30, 40s:Age40, 50s:Age50)
	Marital status	Categorical type (Married:M, Single:N, Divorced/Separated, etc.:E)
	Number of Children	Numerical type (number of children, 0:no children)
	Residential area	Categorical type (Seoul:1, Busan:2, Gyeonggi:3, Incheon:4, Gangwon:5, Chungbuk:6, Chungnam:7, Jeonbuk:8, Jeonnam:9, Gyeongbuk:10, Daegu:11, Gyeongnam:12, Jeju:13, Gwangju:14, Daejeon:15, Ulsan:16, Sejong:17)
	Occupation	Categorical type (blue collar jobs:B, white collar jobs:W, etc.:E)
	Household Monthly Income	Numerical type (amount:KRW)
	Customer Asset Rating Class	Categorical type (high-income professional:1, Office work:2, Production worker of with household monthly income of more than 4millon KRW or Full-time Housewife whose spouse has Office work:3–5, Above 10 million KRW in Monthly income or Above 100 million KRW in Financial assets:6, Above 1 billion KRW in Financial assets:7, Above 3 billion KRW in Financial assets:8, General grade:99)
	Final Education (Insurance Planner)	Categorical type (Graduate school:G, University:U, High school:H, Middle school and below:M)
	Age gap between Customer and Insurance Planner	Categorical type (5 years or more:D, less than 5 years:S)
	Insurance Claim amount	Numerical type (amount:KRW)
	Contract amount at the time of claim	Numerical type (amount:KRW)
	Insurance claim due to cancer disease	Categorical type (No:0, Yes:1)
	Insurance claim due to heart disease	Categorical type (No:0, Yes:1)
	Insurance claim due to brain disease	Categorical type (No:0, Yes:1)
	Insurance claim due to chronic disease	Categorical type (No:0, Yes:1)
	Number of insurance subscriptions	Numerical type (Count)
	Total premium paid	Numerical type (amount:KRW)
	Actual Loss Health Insurance Subscription Status	Categorical type (No:0, Yes:1)

To begin, we considered several demographic and socioeconomic variables that have been analyzed in previous studies to assess their impact on insurance demand and individual risk aversion. The selected variables included gender, age, marital status, number of children, residential region, and occupation. Occupation was classified according to the Korea Insurance Development Institute's categorization and the corresponding "occupational injury risk rating" provided by the insurance company. The grade1 category comprised 214 occupations with low occupational risk, such as office managers and related workers, while the grade2 category consisted of 268 occupations including field managers, restaurant/mart employees, and salespersons. The third category encompassed occupations directly involved in fieldwork, such as construction workers and ship maintenance workers. Insurance companies typically apply different premium rates based on the risk level associated with each occupation. Individuals belonging to grade1 category (low-risk occupations) generally have lower insurance premiums compared to those in the grade2 or grade3 category. This distinction acknowledges the varying levels of injury risk associated with different occupations. In this study, we classified individuals working in office-based roles as belonging to the grade1 category, while individuals engaged in fieldwork were classified as belonging to the grade2 or grade3 category. All other occupational groups were considered as "other."

Furthermore, drawing on findings from previous research that indicate a positive correlation between insurance subscription and consumer income or assets, we included the customer's household monthly income and asset class as additional explanatory variables. The customer asset class was derived from the research target company's high-asset customer rating system. Customers with a monthly income of 10 million won (KRW) or more or financial assets of 100 million KRW or more were considered as potential wealthy individuals, while those with even higher income and assets were classified as wealthy or ultra-wealthy based on the level of their financial holdings. Additionally, high-income professionals, who may not meet the current standards for wealthy individuals but are likely to accumulate substantial assets in the future, were assigned separate ratings (grades 1 to 5). The remaining ratings were categorized as "general" customer grades (99).

While previous studies, such as Dohmen, Falk [44] and Jianakoplos and Bernasek [45], have yielded inconsistent results regarding the effect of education level on risk aversion, we attempted to explore the correlation between an individual's educational background and risk aversion by incorporating it as an explanatory variable. However, the available educational information among the customer data provided by the research target company exhibited a missing value rate of 99.9 %, rendering it unsuitable for analysis. To address this limitation, we opted to utilize the educational background information of insurance solicitors who possess reliable and comprehensive data. Given that insurance products require extensive consideration and comparison before purchase and must be maintained over a long term, the information exchange dynamics between policyholders and insurance solicitors is expected to influence insurance demand. It is hypothesized that the acceptability of information will vary based on the policyholder's level of education. For example, when a highly educated insurance solicitor is involved in a contract, it is assumed that customers with a high rate of insurance subscription would likely possess a higher educational background, as their demand for information tends to be relatively higher.

Additionally, we introduced the age difference between the contracted insurance recruiter and the customer as an explanatory variable. This variable aimed to provide a comprehensive understanding of individual customers' information acceptance, considering the educational background of the insurance recruiter.

Finally, as this study focused on individual customers with a history of illness-related claims, we included several additional explanatory variables to capture their insurance-related behavior. These variables encompassed the amount of claims, contract amount at the time of claim, total amount of paid insurance premiums, and loss subscriptions. Moreover, recognizing that individuals with chronic diseases may exhibit a heightened demand for insurance coverage to mitigate the associated high risks, we incorporated specific diseases as individual explanatory variables. The selected diseases were those deemed relatively severe, such as cancer, heart disease, and brain disease. By referring to the Korean Standard Disease/Sign Classification Table, we applied the criteria for disease names in patients' medical records for the calculation of the research target company's "chronic disease management fee," as presented in Table 5. This inclusion aimed to investigate potential variations in individual risk perception following the occurrence of different disease types and to examine whether such differences manifest in insurance-related decisions. As per this background, the list of variables that are adopted in the current study, as well as the descriptive statistics are presented in Table 6, Table 7.

**Table 5:** Criteria for patients subject to calculation of chronic disease management fees.

Explanatory variable (about Claim disease)	Disease Name (Disease Code) Of ‘Korea Standard Disease/Sign Classification Table’
Chronic disease status (all)	High blood pressure (I10–I13,I15), Diabetes (E10-E14), Mental and Behavioral Disorders (F00–F99,G40-G41), Respiratory Tuberculosis (A15-A16, A19), Nervous System Disease (G00-G37,G43-G83), Malignant Neoplasm (intraepithelial cancer; D00-D09), Disorder of the Thyroid Gland (E00-E07), Liver Disease (B18,B19,K70–K77), Chronic Renal Failure (N18)
Cancer disease status	Malignant Neoplasm (C00–C97)
Heart disease status	Heart disease (I05–I09, I20–I27, I30–I52)
Brain disease status	Cerebrovascular disease (I60–I69)

**Table 6:** Descriptive Statistics for Categorical type Variables (N = 94,306).

Variables	Category	Percentage of each category	Variables	Category	Percentage of each category
Gender	Female	57.4 %	Occupation	Blue collar	34.3 %
	Male	42.6 %		White collar	39.7 %
Age	Under30s	27.5 %		Etc.	26.1 %
	30s	30.9 %	Customer Asset Rating Class	1	18.5
	40s	26.5 %		2	23.8
	50s	15.2 %		3∼5	12.8
Marital status	Married	36.4 %		6	14.2
	Single	26.8 %		7	1.3
	Divorced/Separated	0.3 %		8	0.3
	etc.	1.7 %		General	25.6
	null (missing value)	34.6 %		null (missing value)	3.5 %
Residential area	Metropolitan (Seoul, Gyeonggi, Incheon)	51.7 %	Final Education (Insurance Planner)	Graduate school	0.2 %
	Local large city (Busan, Ulsan, Daegu, Daejeon, Sejong, Gwangju)	17.7 %		University	11.6 %
	Regional area Gyeongnam, Gyeongbuk, Gangwon, Chungbuk, Chungnam, Jeonbuk, Jeonnam, Jeju)	30.6 %		High school	18.2 %
Insurance claim due to cancer disease	No	97.8 %		Middle school and below	0.03 %
				null (missing value)	70.0 %
	Yes	2.2 %			
Insurance claim due to heart disease	No	99.3 %	Age gap between Customer and Insurance Planner	5 years or more	94.0 %
	Yes	0.7 %		less than 5 years	6.0 %
Insurance claim due to brain disease	No	99.4 %	Actual Loss Health Insurance Subscription Status	No	32.2 %
	Yes	0.6 %		Yes	67.8 %
Insurance claim due to chronic disease	No	90.4 %	–	–	–
	Yes	9.6 %	–	–	–

**Table 7:** Descriptive Statistics for Numeric type Variables (N = 94,306).

Variables	Min	Max	Median	Mean	Dispersion
Number of Children	0	10	0	0.67	1.39
Household monthly income (amount:1000 KRW)	0	100,000,000	3000	10,426	66.77
Insurance Claim amount (amount:1000 KRW)	0.1	10,000	300	911.94	2.02
Contract amount at the time of claim (amount:1000 KRW)	150,000	1,000,000	340,000	384,116.57	0.47
Number of insurance subscription (count)	1	10	2	2.34	0.79
Total Premium paid. (amount:1000 KRW)	1.32	10,000	207	526.79	2.37

### 3.2 Creation of predictive model

In this study, we aimed to develop a machine learning-based insurance product additional subscription prediction model to objectively investigate the individual characteristics that influence risk avoidance tendencies. To achieve this, we employed seven different machine learning algorithms: Logistic Regression, Naive Bayes, Decision Tree, Random Forest, Gradient Boosting, SVM, and Neural Network.

Logistic regression (LR) is commonly used for discrete data categorization, modeling with an S-shaped curve to provide probabilistic predictions. This grants interpretability but may perform less effectively with non-linear patterns and complex relationships.

The Naïve Bayes model employs a straightforward Bayesian network approach, where class variables are independently assigned to all attributes. It utilizes a simple supervised learning method that calculates a combination of frequencies and values within the given dataset. Therefore, fast and accurate prediction is possible even when using a large amount of data, but the assumption of conditional independence that all features must be independent of each other is difficult to establish in reality.

Decision trees, a widely used analytical method, utilize tree structures to define decision rules for categorizing populations or predictive modeling. They are favored for their interpretability and visual clarity, requiring less preprocessing and handling non-linear relationships. However, a drawback is their tendency to create complex, overfit models sensitive to data fluctuations.

Random Forest, a machine learning ensemble method, addresses limitations in decision trees, particularly overfitting. It builds multiple tree models using bootstrapped samples from the training data, introducing randomness to create uncorrelated trees. This boosts predictive power and stability while mitigating overfitting, thanks to the law of large numbers.

Support Vector Machine (SVM) is a binary classifier that maps data into a high-dimensional feature space and determines an optimal decision boundary for categorization. It employs structural risk minimization (SRM) to avoid overfitting and achieve global optima. However, SVMs are less interpretable due to their black-box nature, have longer learning times, and require data normalization for convenience.

Gradient Boosting Models (GBM) employ a more sophisticated prediction technique by iteratively weighting and combining a series of weak learners in a gradual and sequential manner, thus enhancing performance. However, it is worth noting that GBM models carry the risk of overfitting when trained on data contaminated with noise.

Deep learning neural networks predict data by employing feed-forward connections between input, hidden, and output layers composed of complex neurons linked by synapses. While they excel in analyzing intricate models, their analysis times can be exceedingly long. As model complexity grows, analysis becomes computationally challenging. These networks are often seen as black-box models, and data scaling like normalization is necessary, akin to SVM models.

These algorithms were implemented using the open-source software Orange version 3.34.0, which is a Python-based toolkit for data visualization, machine learning, and data mining. Orange provides a visual programming front end for exploratory data analysis and interactive data visualization and was used as the analysis tool to develop the predictive model in this study.

To enhance the reliability of the machine learning prediction model, we divided the variables into a training set and a testing set to verify the performance of the prediction model. Random Sampling and K-fold Cross Validation were used to extract the training set.

Random Sampling, an in-sample testing technique, assesses the predictive capability of training data by randomly splitting it into training and test sets. In this study, the training set comprised 66.66 % of the data, with the remaining 33.33 % allocated to the test set. This random division process was repeated five times.

Conversely, K-fold Cross Validation represents an out-of-sample testing approach that assesses a model's ability to predict the dependent variable in validation data. It partitions the dataset into K subsets, using one for validation and the remainder for training, repeating this process K times and averaging the performance scores. In this analysis, we segmented the variables into five distinct subsamples (1, 2, 3, 4, and 5) and iterated the estimation procedure five times. Each iteration employed a different subsample for validation, while the others constituted the training set. The utilization of K-fold cross-validation offers the benefit of ensuring that all data serves as validation at least once, thus safeguarding against biased performance assessment and enhancing model performance validation. However, it is worth noting that this approach may demand significant computational resources and time due to the evaluation of multiple models across the folds.

The computational time needed may vary based on the researcher's computing environment. In our analysis, we assessed the performance of seven algorithms under consistent conditions (CPU: Apple M2, RAM: 8 GB). We observed that the random sampling method required 7 min and 10 s, while the k-fold cross-validation method demanded 10 min and 25 s, resulting in a time difference of under 3 min. However, the performance evaluation of the seven algorithms indicated that random sampling yielded an average accuracy of 0.740, whereas k-fold cross-validation achieved an average accuracy of 0.748. This demonstrates a slight enhancement in prediction reliability when employing the k-fold cross-validation method. Consequently, our study aimed to enhance the predictive model's reliability through the utilization of k-fold cross-validation, which provides a more precise measure of model quality without significantly prolonging the validation process.

In order to determine the best-performing model among the seven machine learning algorithms, we evaluated them based on Classification Accuracy, F1-score, and Area Under the Curve (AUC). Classification Accuracy measures the ratio of correctly predicting actual "additional subscriptions" and "no additional subscriptions." F1-score is the harmonic average of recall and precision and indicates the balance between the ability to correctly predict positive cases and the ability to avoid false positives. AUC represents the area under the Receiver Operating Characteristic (ROC) curve, which plots the false positive rate against the true positive rate. A higher AUC indicates better model accuracy [50].

By assessing these performance indicators, we selected the top-performing models and treated them as the Champion models for further analysis. Additionally, preprocessing steps were executed to handle missing data in these two models. Following an examination of the missing value patterns within the independent variables, we formulated appropriate strategies. This encompassed the removal of rows containing missing values for customer asset grade and marital status variables, as our analysis revealed that these missing values could not be effectively substituted with other explanatory variables.

Furthermore, we explored the possibility of improving performance compared to individual models by combining the best-performing Champion models. This approach, known as stacking, aims to create an ensemble model that leverages the strengths of different models and mitigates overfitting issues while reducing biases [51]. In this study, we combined the final two best-performing single models into a stacking ensemble model to create a predictive model that capitalizes on the advantages of each model. We then compared the predictive performance of the individual models and the stacking model to assess their relative effectiveness.

### 3.3 Predictive determinants analysis

To assess the stability and reliability of the developed predictive model for additional insurance coverage, as well as to identify the factors that determine such coverage even after claiming insurance due to disease, the importance of key predictive determinants was derived.

The contribution of explanatory variables to the performance of the predictive model for additional insurance products was evaluated using the ReliefF (Regional ReliefF) algorithm, which belongs to the family of relief algorithms and is a filter method in machine learning. This algorithm captures both linear and non-linear interactions between features and the target variable by assigning scores based on the concept of closest neighbors. High-scoring instances are then extracted and utilized for machine learning predictions [52]. The original relief algorithm measures the quality of a property based on how effectively values distinguish between closely related instances. In the case of RReliefF, further enhancements were made by incorporating probability functions to determine whether two instances differ and calculating the weight of each function based on this probability value [53]. In this study, the RReliefF algorithm was implemented using Orange 3.34.0.

Finally, a comparative and analytical examination was conducted to explore the impact of each explanatory variable's detailed characteristics on the target value. By scrutinizing the specific characteristics associated with individuals predicted to exhibit a propensity for additional insurance coverage, a diagram illustrating these characteristics was presented.

This comprehensive analysis aimed to provide insights into the factors that significantly influence the likelihood of individuals opting for additional insurance coverage, even after experiencing insurance claims related to illness.

## 4 Results

### 4.1 Prediction of additional insurance subscriptions

As depicted in Table 8, the evaluation of the seven machine learning algorithms utilized in this study revealed promising results. Specifically, the training and test set performance of five algorithms, namely Gradient Boosting, Logistic Regression, Neural Network, Random Forest, and Naïve Bayes, demonstrated a remarkable average accuracy exceeding 0.75. This implies that the model, incorporating 19 variables encompassing personal characteristics, contracts, and55] claims as explanatory factors, can predict the probability of individuals acquiring additional life insurance products with an accuracy of more than 75 % within a span of approximately 1–2 years following a disease-related insurance claim.

**Table 8:** Evaluation score comparison of the seven algorithm test results.

Model	AUC	CA	F1	Precision	Recall
Tree	0.517	0.702	0.696	0.690	0.702
SVM	0.468	0.664	0.661	0.657	0.664
Random Forest	0.619	0.776	0.720	0.711	0.776
Neural Network	0.642	0.781	0.727	0.725	0.781
**Naïve Bayes**	**0.648**	**0.755**	**0.733**	**0.720**	**0.755**
Logistic Regression	0.459	0.788	0.695	0.762	0.788
**Gradient Boosting**	**0.675**	**0.789**	**0.709**	**0.741**	**0.789**

Assessing the model's efficacy using F1-scores, the Naïve Bayes algorithm achieved a score of 0.733, slightly outperforming the Neural Network (0.727), Random Forest (0.720), Gradient Boosting (0.709), and Logistic Regression (0.695) algorithms.

Furthermore, the analysis of Area Under the Curve (AUC) values indicated that Gradient Boosting attained the highest AUC score at 0.675. Naïve Bayes, Neural Network, and Random Forest followed closely with AUC values of 0.648, 0.642, and 0.619, respectively. However, the AUC value for Logistic Regression stood at 0.459, falling short of the minimum standard required for robust model evaluation.

Consequently, considering the high accuracy, F1-score, and AUC values, the Naïve Bayes and Gradient Boosting algorithms were selected as the Champion model, exhibiting superior predictive power compared to the other algorithms.

To mitigate the issues of conditional independence and overfitting associated with the Naïve Bayes and GBM models, a Stacking Ensemble model was created by combining these two models. The predictive performance of this ensemble model was then compared against the individual Naïve Bayes and Gradient Boosting models.

Additionally, during the data input stage, certain explanatory variables such as marital status and customer asset rating contained missing values. To address this, categorical variables associated with missing values were treated by excluding the corresponding personal data (rows) from the analysis. The number of cases with missing values in marital status amounted to 32,670, accounting for 34.6 % of the total 94,306 cases, while the number of missing data points in the customer asset rating was 3,307, representing 4 % of the total.

As demonstrated in Table 9, the Stacking Ensemble model, implemented under the same conditions as the Naïve Bayes and Gradient Boosting models in this analysis, yielded improved predictive performance with an accuracy of 0.789, F1-score of 0.720, and AUC of 0.675. Notably, these results outperformed the Naïve Bayes model, which achieved an accuracy of 0.756 and an AUC of 0.651. Although the accuracy and AUC of the Stacking Ensemble model were comparable to Gradient Boosting, the F1-score of the ensemble model (0.720) surpassed that of Gradient Boosting (0.708).

**Table 9:** Evaluation score comparison of test results for stacking ensemble algorithms.

Model	AUC	CA	F1	Precision	Recall
**Stack**	**0.675**	**0.789**	**0.720**	**0.738**	**0.789**
Naïve Bayes	0.651	0.756	0.734	0.721	0.756
Gradient Boosting	0.675	0.789	0.708	0.742	0.789

In conclusion, leveraging the Stacking Ensemble model, which demonstrated superior predictive power, the study successfully predicted the subscription status of additional insurance products for each individual.

### 4.2 Analysis of factors affecting risk aversion

The insurance product addition prediction model analyzed a total of 19 explanatory variables categorized into socioeconomic characteristics, demographic characteristics, health/disease status, and insurance product acceptance. The objective was to identify risk-averse individual characteristics among customers who had claimed insurance due to disease. The analysis aimed to determine which independent variables contained the most information for predicting additional insurance product subscriptions, reflecting risk aversion tendencies.

Using the RReliefF (Regressional ReliefF) algorithm, the contribution of each explanatory variable to the predictive model's performance was evaluated. As depicted in Table 10, The top 10 variables were selected based on their contribution, shedding light on the relationship between each variable's characteristics and individual risk aversion. The results revealed the most important variable for predicting risk aversion to be the residential area (ReliefF value: 0.048), followed by customer asset rating (ReliefF value: 0.044), occupational group (ReliefF value: 0.028), and age group (ReliefF value: 0.22). These top four variables played a significant role in predicting risk aversion, out of the 19 independent variables. Consequently, the variables ranking from 11th to 19th in ReliefF (e.g., total payment insurance premiums, claims for brain diseases) were deemed insignificant in identifying individual risk aversion tendencies and were excluded from the predictive model.

**Table 10:** Importance of Factors influencing additional insurance subscriptions (ReliefF value).

	Factor	Rank	ReliefF
Demographic Characteristics	**Residence**	**1**	**0.048**
	**Age**	**4**	**0.022**
	**Marital status**	**9**	**0.012**
	**Gender**	**10**	**0.010**
	Number of Children	13	0.004
Socioeconomic Characteristics	**Customer Asset rating class**	**2**	**0.044**
	**Occupation**	**3**	**0.028**
	**Final Education (Insurance Planner)**	**5**	**0.022**
	Age gap (Customer-Insurance Planner)	15	0.002
	Household Monthly Income	16	0.000
Health Characteristics	**Insurance claim due to Cancer disease**	**8**	**0.014**
	Insurance Claim Amount	11	0.009
	Insurance claim due to Chronic disease	12	0.006
	Insurance claim due to Heart disease	17	0.000
	Insurance claim due to Brain disease	18	0.000
Acceptance of Insurance Product	**Actual Loss Health Insurance Subscription Status**	**6**	**0.020**
	**Contract amount at the time of claim**	**7**	**0.019**
	Number of insurance subscriptions	14	0.003
	Total premium paid	19	−0.002

Regarding demographic characteristics, factors such as residence, age group, marital status, and gender showed a relatively greater influence on individuals’ inclination to avoid risks through additional insurance product subscriptions.

**Fig. 1:**
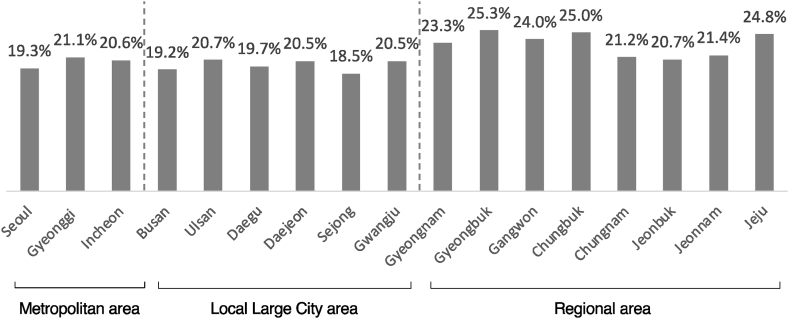
Comparative Analysis of the Share of Additional Insurance Subscription Predictors by Residential area.

For instance, Fig. 1 below displayed the share of additional insurance product subscribers by residential area. The metropolitan and large city areas of Korea (Seoul, Gyeonggi, Incheon, Busan, Ulsan, Daegu, Daejeon, Sejong, Gwangju) had an average share of 20.2 %, which was 3.1% points lower than the regional average of 23.3 % (Gyeongnam, Gyeongbuk, Gangwon, Chungbuk, Chungnam, Jeonbuk, Jeonnam, Jeju). This indicates that residents of metropolitan and large city areas have a higher propensity for risk compared to rural areas. In the comparison of additional subscription predictors between individuals residing in large cities and those residing in nearby provinces, the share of predictors for residents in metropolitan areas was relatively low, implying that urban areas exhibit a preference for risk over rural areas. (Seoul19.3 % vs Gyeonggi21.1 %, Busan19.2 %&Ulsan20.7 % vs Gyeongnam23.3 %, Daegu19.7 % vs Gyeongbuk25.3 %, Daejeon20.5 %&Sejong18.5 % vs Chungnam21.2 %&Chungbuk25.0 %, Gwangju20.5 % vs Jeonnam21.4 %)

**Fig. 2:**
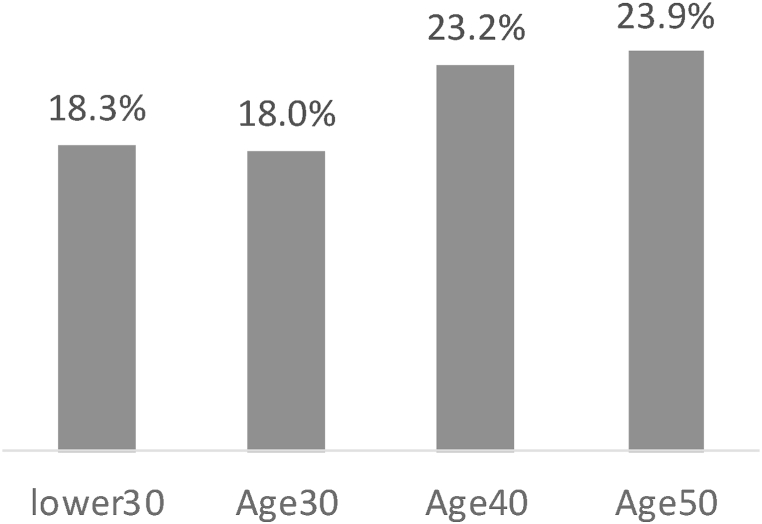
Comparative analysis of the share of additional insurance subscription predictors by age.

Fig. 2 displayed the share of additional insurance subscription predictors by age group. It showed that the share increased with age, with 18.3 % for individuals in their 20s and younger, 18.0 % for those in their 30s, 23.2 % for those in their 40s, and 23.9 % for those in their 50s. This suggests that risk aversion tends to rise as individuals grow older, possibly due to the decreasing time available for recovering losses resulting from risks.

**Fig. 3:**
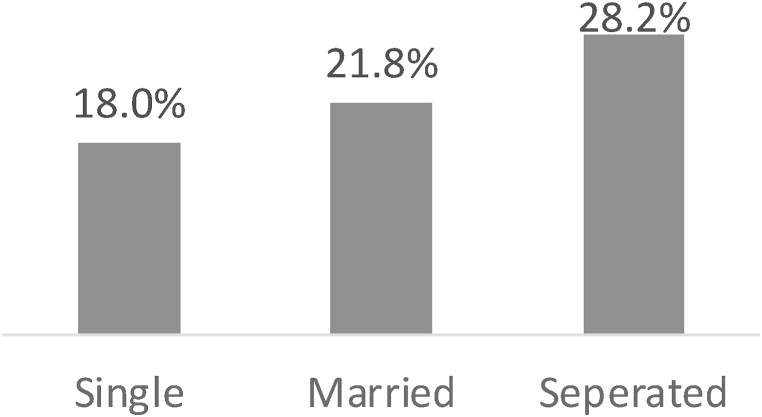
Comparative analysis of the share of additional insurance subscription predictors by marital status.

Marital status also played a role in risk aversion tendencies. As depicted in Fig. 3, the share of additional insurance product predictors was 18.0 % for single individuals, 21.8 % for married individuals, and 28.2 % for separated individuals. This indicates that married individuals tend to be more risk averse than single individuals, while those who are separated due to divorce or bereavement exhibit even higher risk aversion.

Furthermore, the share of additional insurance product predictors by gender was 22.1 % for women and 19.9 % for men, illustrating that women have a stronger tendency toward risk aversion compared to men.

The factors identified as important variables for predicting risk aversion among the factors related to individual socio-economic characteristics were customer asset rating, occupational group, and financial planner's level of education.

**Fig. 4:**
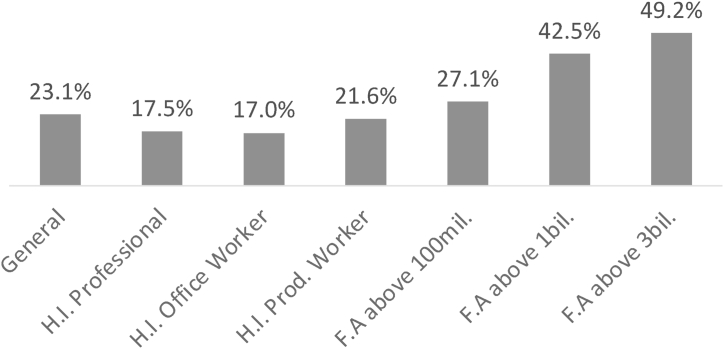
Comparative Analysis of the Share of Additional Insurance Subscription Predictors by Customer Asset Rating Class. note) H.I. indicates High Income, F.A indicates Financial Asset.

Examining the customer asset rating, Fig. 4 revealed that the share of additional subscription predictors increased with the size of financial assets held. For instance, it was 27.1 % for individuals with financial assets of 100 million or more, 42.5 % for those with financial assets of 1 billion or more, and 49.2 % for those with financial assets of 3 billion or more, all higher than the 23.1 % for the general grade. This suggests a positive correlation between the size of financial assets and risk aversion tendencies, same as confirmed in previous studies of Xiao [6], Bernheim [7] and Hau [8]. Moreover, the share of additional subscription predictors for high-income professionals was 17.5 %, higher than the 17.0 % for high-income office workers. This may be due to the relatively higher risk of unemployment faced by office workers compared to high-income professionals, resulting in a lower demand for insurance subscriptions. This is consistent with previous studies by Kagraoka [14] and Kiesenbauer [13] confirming that there is a negative correlation between the unemployment rate and the insurance subscription retention rate.

However, the share of predictors of additional insurance products for high-income production workers was 21.6 %, which was relatively high compared to high-income professional and high-income office workers. This is the same as the “injury risk rating by occupation” disclosed by the Korea Insurance Development Institute, where office and professional occupations with low job risk have a lower risk of injury than field and production occupations with relatively high job risk. In other words, it can be judged that a person with a production job group has a higher risk aversion tendency because they are exposed to a relatively high-risk environment for vocational activities.

Similar patterns were observed when considering occupation groups. Production workers exhibited a higher risk aversion tendency compared to office workers, as evidenced by the share of blue-collar predictors (25.6 %) being significantly higher than that of white-collar predictors (18.5 %).

Among the socio-economic characteristics, the educational background of insurance planners emerged as an important variable (ReliefF value: 0.022) ranking 5th in the ReliefF ranking. When insurance planners had a high school education or lower, the share of additional subscription predictors among total subscribers was 23.5 %, while it was 21.1 % when the financial planner held a university degree or higher. This implies that customers of insurance products exhibit a preference for risk when insurance agents and policyholders share a balanced level of education. 10.13039/100014337Furthermore, a survey conducted by the Korea Life Insurance Association revealed a correlation between the level of education of the head of the household and the recognition rate of life anxiety, supporting the findings of this study. It was found that the lower the level of education by householder, the higher the recognition rate of life anxiety, as shown in Table 11.

**Table 11:** Awareness of Life Anxiety (Survey of the Korea Life Insurance Association, n = 2000).

		Number of cases	Recognition rate of life anxiety (%)
Total		(2000)	36.4
highest level of education of head of household's	Elementary school graduation or less	(84)	59.0
	Middle School	(93)	57.2
	High School	(718)	43.9
	University graduate or higher	(1105)	28.1

Because health was identified as the most common cause of life anxiety, as indicated by the Korea Life Insurance Association's survey, this study focused on individuals with a history of claiming insurance due to disease. Within this context, claims for reasons related to cancer disease emerged as an important variable (ReliefF value: 0.014) for predicting risk aversion. Individuals with a history of cancer-related claims displayed a relatively high propensity for risk aversion through additional insurance subscriptions. However, variables such as claim amount, claim for chronic disease, claim for heart disease, and claim for brain disease had limited impact on the predictive model for additional insurance subscriptions, likely due to individuals with a history of disease being frequently denied insurance subscriptions during the review process.

**Fig. 5:**
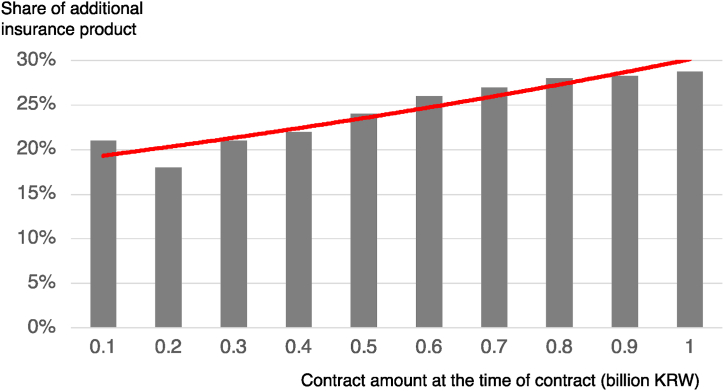
Comparative Analysis of the Share of Additional Insurance Subscription Predictors based on Contract amount at the Time of Claim.

Lastly, among the factors related to insurance product acceptance, whether to sign up for loss (ReliefF value: 0.020) and the contract amount at the time of claim (ReliefF value: 0.019) ranked 6th and 7th, respectively, in the ReliefF ranking. These factors exerted a significant influence on the prediction of additional insurance subscriptions. Fig. 5 demonstrated that a larger insurance contract amount at the time of claim corresponded to a higher proportion of subscribers expected to sign up for additional insurance. This aligns with previous studies on insurance demand theory, indicating that risk-averse individuals tend to increase their insurance demand. Furthermore, individuals with a perception of greater risk attempt to mitigate it through the purchase of insurance, and this risk aversion tendency persists even after a claim is made.

**Fig. 6:**
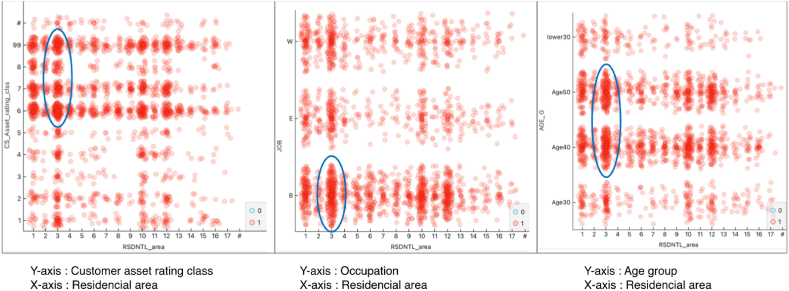
Scatter Plot of Predictor variable (example: Case when both actual and predicted values are ‘Yes’).

Furthermore, amalgamating the top 10 variables proven to impact both the demand for supplementary insurance and individuals' risk-averse tendencies facilitates a more holistic comprehension of individual attributes. For instance, as exemplified in Fig. 6 below, when we scrutinize the scatter plot illustrating predicted versus actual additional insurance acquisitions concerning explanatory variables such as residential area, customer asset class, occupation, and age, it becomes evident that individuals in their 40s and 50s, employed in productive roles, residing in the Gyeonggi region (residential area characteristic '3′), and possessing financial assets exceeding 100 million KRW (customer asset rating class ‘6’ or higher), exhibit a heightened proclivity toward risk aversion. Consequently, they are more inclined to engage in additional insurance purchases.

## 5 Discussions and implications

The insurance economics theory suggests that the demand for insurance increases with higher risk aversion tendencies. Previous studies attempting to empirically validate this theory faced limitations in quantitatively measuring risk aversion, a psychological variable of individuals, through survey methods. As a result, efforts were made to establish a correlation between demographic and socioeconomic variables, such as gender, income, education level, and marital status, and insurance demand. However, the consistency of the correlation between these variables and insurance demand was inconclusive, as revealed in previous research studies. Thus, it was challenging to conclude that questionnaire items accurately reflected individuals' risk aversion tendencies.

In contrast, this research empirically examines the theory of insurance demand, positing that greater risk aversion corresponds to an increased inclination for securing comprehensive life insurance encompassing protection against illness, injury, and mortality. Leveraging extensive data acquired from a prominent Korean life insurance provider, this study employed 19 independent variables encompassing individual demographic attributes, socioeconomic status, insurance agreements, and claims records. The dependent variable under scrutiny was the decision to acquire supplementary insurance products. Utilizing a predictive model, we employed the most effective algorithm among seven machine learning techniques to formulate our analysis.

Through this study, it was confirmed that the level of risk aversion, measured by the additional insurance subscriptions of individuals who had a heightened risk perception due to diseases, could be accurately predicted using variables reflecting the individual's actual risk aversion. Thus, it can be concluded that the variables related to risk aversion propensity appropriately capture each individual's inherent risk aversion.

We further assessed the significance of variables to identify the top 10 characteristic factors with the most pronounced influence on an individual's inherent risk aversion. Subsequently, we conducted a correlation analysis between these factors and an individual's level of risk aversion.

Summarizing the risk aversion characteristics for each of the top 10 variables with significant impact on the predictive model, they appear as in Table 12. The findings are as follows: Rural residents are more risk-averse than city dwellers. Women tend to be more risk-averse than men. Individuals with higher financial assets exhibit higher risk aversion tendencies. Blue-collar workers are more risk-averse than white-collar workers. Lower educational attainment is associated with higher risk aversion. Married individuals are more risk-averse than unmarried individuals, except in cases where marital status changes due to reasons like divorce or bereavement. Risk aversion is more pronounced among individuals with a history of cancer-related claims. Individuals with larger contract amounts and indemnity insurance show a greater risk aversion propensity. The willingness to sign up for actual loss coverage contributes significantly to predicting additional insurance purchases. The type and amount of insurance coverage at the time of claim influence risk aversion and subsequent additional insurance purchases.

**Table 12:** Correlations between explanatory variables and risk aversion.

Prediction importance ranking	Explanatory variable	Correlation of Risk Aversion Propensity
1	Residence	Rural areas (+), Urban areas (−)
2	Customer Asset rating class	Asset size (+)
3	Occupation	Blue collar (+), white collar (−)
4	Age	Older age (+)
5	Final Education (Insurance Planner)	Low education (+)
6	Actual Loss Health Insurance Subscription Status	Subscription (+)
7	Contract amount at the time of claim	Large amount (+)
8	Insurance claim due to Cancer disease	Claim due to cancer disease (+)
9	Marital status	Separated (+) > Married (+) > Single (−)
10	Gender	Female (+), male (−)

Note: (+) Positive of Insurance subscription demand; risk aversion, (−) Negative of Insurance subscription demand; risk preference.

In contrast, this study provides a quantitative assessment of individuals' risk aversion by analyzing substantial real-world data. In comparison, prior research examined individuals' risk aversion qualitatively through survey methods. The outcomes of both approaches are summarized in Table 13.

**Table 13:** Comparison between the results of prior research and this study.

	Influence Factor	Previous Study		This Study	
		Reference	Corelation	Corelation	Matching
Demographic characteristics	Age	Berekson [16], Showers and Shotick [48], Gutter and Hatcher [49], Kim&Jung [1]	+	+	O
		Ferber and Lee [47]	–		X
		Hammond, Houston [36]	n.s		X
	Gender	Joung and Shin [23], Kim&Jung [1]	Female > male	Female > male	O
	Marital Status	Hammond, Houston [36], Joung and Shin [23]	Married > single	Separated >married >single	O
		Berekson [16]	n.s		X
	Residence	Lee, Kwon [41]	Rural > urban	Rural > urban	O
Socioeconomic characteristics	Assets held	Bernheim [7], Xiao [6], Hau [8]	+	+	O
		Di Matteo and Emery [17]	–		X
		Berekson [16], Anderson and Nevin [15]	n.s		X
	Occupation	Lee, Kwon [41]	Blue collar > white collar	Blue collar > white collar	O
	Education level	Gutter and Hatcher [49], Dohmen, Falk [44], Outreville [46]	–	–	O
		Hammond, Houston [36], Ferber and Lee [47], Jianakoplos and Bernasek [45]	+		X
Health Status	Insurance claim due to Cancer disease	Kim [43]	+	+	O
Insurance Preference	Actual Loss Insurance Subscription Status	Yaari [27], Hakansson [28]	+	+	O
	Contract amount at the time of claim	Yaari [27], Hakansson [28]	+	+	O

Note: (+) Positive of Insurance subscription demand; risk aversion, (−) Negative of Insurance subscription demand; risk preference n.s indicates the statistical insignificance.

Regarding marital status, the findings were consistent with previous studies, such as Hammond, Houston [36] suggesting that individuals in married status exhibit higher risk aversion compared to unmarried individuals. The increased responsibility and consideration for the welfare of both spouses may contribute to married individuals feeling a greater psychological burden regarding potential risks. Notably, this study added new dimensions by including cases of marital life disruption, such as divorce or bereavement, as influencing factors and examining their impact on risk aversion. It was observed that divorced (including bereaved) individuals displayed stricter risk aversion tendencies compared to married individuals. This could be attributed to the greater psychological burden faced by married individuals, including financial losses such as a decrease in household income, as their marriage ends. In fact, according to a 2022 survey by the National Statistical Office of Korea, the share of double-income households among married households reached a record high of 46.3 %, with double-income households having an average monthly income 1.58 times higher than single-income households.

On contrast, the results of this study aligned with previous research, demonstrating consistent effects of variables such as gender, residential area, occupation, education level, claims related to cancer diseases, indemnity insurance, and the amount of existing insurance contracts on the demand for additional insurance. For instance, men exhibited a lower probability of subscribing to additional insurance compared to women, indicating a higher risk-taking propensity among men. This finding not only concurred with several previous international studies but also demonstrated the gender effect on risk aversion among Korean consumers, as investigated by Joung and Shin [23].

Regarding residential area, this study, similar to the research conducted by Lee, Kwon [41], examined the impact of residential area and job classification on insurance demand. The findings were consistent with previous studies, suggesting that consumers living in rural areas display higher risk aversion and a greater demand for additional insurance. However, it is important to consider that consumers residing in urban areas, with relatively higher employment opportunities, may not exhibit a significantly high demand for additional insurance, as they often benefit from health insurance provided by their workplaces. On the other hand, individuals living in rural areas, where cooperative relationships such as blood ties are prevalent due to the agricultural nature of the region, may find it easier to be influenced by insurance solicitors, thereby contributing to higher sales in rural areas. Hence, adopting a multidimensional approach becomes crucial when assessing an individual's risk aversion tendency based on their residential area.

Concerning occupation, the results of this study aligned with previous research, indicating that individuals with blue-collar jobs tend to have a higher risk aversion propensity compared to those with white-collar jobs. This observation suggests that consumers consider the differences in risk occurrence possibilities based on their occupations. For instance, the Korea Insurance Development Institute's "injury risk rating by job" highlights that office workers with lower risk ratings (grade 1) face lower job risks compared to production workers and field workers with higher risk ratings (grades 2 and 3).

Similar to the study conducted by Kim [43], this research identified the significance of whether or not a claim related to cancer disease was made as a variable affecting the prediction of additional insurance product subscriptions, depending on the type of claimable disease. This could be attributed to the heightened risk perception among Korean consumers concerning income loss due to medical expenses and job loss for cancer patients. The unique situation in Korea, where cancer incidence and mortality rates are high, with cancer being the leading cause of death and three times higher than the second cause of heart disease (Korea Statistics Office 2013), likely contributes to this perception. To improve customer retention rates, Korean life insurance companies should offer insurance products that alleviate the direct and indirect burdens associated with cancer treatment. Moreover, policy efforts should focus on encouraging low-risk individuals to subscribe to additional cancer insurance products.

## 6 Conclusion

This study employed a novel approach by utilizing a comprehensive real-world dataset that provided a significant advantage over previous studies, which relied on survey methods with limited sample sizes and potential biases.

In conclusion, this study made significant contributions by analyzing the relationship between risk aversion and insurance demand using big data. The findings highlighted the importance of consumer demographics and socioeconomic factors in understanding subjective and psychological risk aversion. Therefore, these findings hold practical significance for insurers seeking to attract and retain customers. By predicting an individual's risk aversion and customizing insurance products to align with each consumer's preferences, insurers can enhance their customer engagement and satisfaction levels.

The model introduced in this research empowers insurers to discern and focus on risk-averse consumers who exhibit a higher likelihood of acquiring additional insurance. By tailoring personalized offers for additional products to these individuals, insurers can effectively nurture customer relationships. Consequently, this model proves beneficial for insurers aiming to enhance customer retention. Brockett and Golden [56] uncovered a positive correlation between the medical loss ratio (MLR) and health improvement efficiency (HealthImpEff) among Medicare Advantage insurers. Hence, when targeting specific individuals based on their personal attributes to encourage insurance purchases, we can anticipate increased efficiency in the medical loss ratio aligned with the insurer's strategic objectives. This is due to the selective allocation of medical cost expenditure for the targeted individuals, thereby maximizing functional health improvements among this demographic.

Additionally, this study holds academic significance as it quantitatively elucidates the personal attribute of 'risk aversion,' a feat previously unattainable. It effectively surmounts the limitations of prior research and underscores the importance of variables influencing an individual's risk aversion by analyzing the profiles of individuals predicted to purchase supplementary insurance products through extensive data analysis employing machine learning techniques. In conclusion, our research successfully delineated the impact of factors such as age, marital status, asset holdings, and residential location on risk aversion. Notably, the finding that residential location stands as the most influential variable in additional insurance purchase, particularly given insurers' access to precise geographic data like zip codes and urban/rural distinctions [54], imparts valuable insights. These characteristics pertaining to individual risk aversion hold potential relevance across diverse fields beyond insurance demand prediction, spanning business administration, psychology, education, and sociology.

However, it is important to acknowledge that satisfaction with product services can also wield significant influence on product enrollment, as evidenced by Brockett and Golden's study [57], which revealed that inadequate service quality can lead to insurance cancellations. Nevertheless, it's essential to acknowledge a limitation of this study, which is the absence of the variable measuring satisfaction with the insurance product. Consequently, future research endeavors should seek to comprehensively discern risk-averse traits within the general population. This can be accomplished by employing the logical structure of the predictive model utilized in our study to incorporate various cognitive variables, including satisfaction with an individual's insurance product, into the decision-making process for purchasing new insurance products.

Additionally, it is important to acknowledge the limitations of this study, which focused on existing insurance customers and may have inherent biases in the characteristics of the research data, favoring individuals with a relatively higher risk-averse tendency compared to non-insurance purchasers. To address these limitations, future research should aim to secure data on general individuals who do not subscribe to insurance products, allowing for a more comprehensive analysis.

While risk aversion constitutes a pivotal and extensively explored aspect within insurance demand theory, it's worth noting that this study is constrained by its reliance on data reflecting the traits of insurance consumers in Korea exclusively. Subsequent research endeavors should aspire to comprehensively gauge the universal association between insurance demand and risk aversion. To achieve this, additional data encompassing dimensions such as religion, hobbies, medical behaviors, and leisure activities should be objectively incorporated. This holistic approach will enable a more precise assessment of the psychological risk aversion levels among insurance consumers, not only within Korea but also across the East Asian region, and potentially extending to Europe and the Americas. Such endeavors will facilitate the identification of fitting strategies and solutions.

## Ethic statement

Review and/or approval by an ethics committee was not needed for this study because anonymized data were used for the academic purpose only, and no experiments with human participants or animals were performed by authors.

## Data availability statement

The data are not publicly available due to their containing information that the authors do not have permission to share. However, the data supporting this study's findings are anonymized and available on request from the corresponding author upon reasonable request for non-commercial purpose.

## Funding statement

This research did not receive any specific grant from funding agencies in the public, commercial, or not-for-profit sectors.

## Additional information

No additional information is available for this paper.

## CRediT authorship contribution statement

**Sehyun Lim:** Conceptualization, Data curation, Formal analysis, Methodology, Project administration, Validation, Writing – original draft, Writing – review & editing. **Taeyeon Oh:** Data curation, Formal analysis, Methodology, Validation, Writing – original draft, Writing – review & editing. **Guy Ngayo:** Conceptualization, Methodology, Writing – review & editing.

## Declaration of generative AI and AI-assisted technologies in the writing process

During the preparation of this work the author(s) used ChatGPT in order to check the grammar and typos. After using this tool/service, the author(s) reviewed and edited the content as needed and take(s) full responsibility for the content of the publication.

## Declaration of competing interest

The authors declare that they have no known competing financial interests or personal relationships that could have appeared to influence the work reported in this paper.
